# *In vitro* and *in silico* evaluation of *Ononis* isoflavonoids as molecules targeting the central nervous system

**DOI:** 10.1371/journal.pone.0265639

**Published:** 2022-03-17

**Authors:** Nóra Gampe, Dominika Noémi Dávid, Krisztina Takács-Novák, Anders Backlund, Szabolcs Béni

**Affiliations:** 1 Department of Pharmacognosy, Semmelweis University, Budapest, Hungary; 2 Department of Pharmaceutical Chemistry, Semmelweis University, Budapest, Hungary; 3 Department of Pharmaceutical Biosciences, Pharmacognosy, Uppsala University, Uppsala, Sweden; Hungarian Academy of Sciences, HUNGARY

## Abstract

Isoflavonoids with various structural elements show a promising potential effect on central nervous system activities. Despite their favorable medicinal properties, the pharmacokinetic characteristics of this thoroughly investigated group of natural phenolics have only been described to a limited extent. Regarding the lack of information about the BBB permeability of isoflavones, isoflavanones, and pterocarpans found in *Ononis* species, the aim of our study was to investigate their physico-chemical properties influencing their absorption and distribution. Furthermore, we aimed to characterize the possible MAO-B inhibiting features of *Ononis* isoflavonoids *in silico*. Octanol-water partitioning and BBB-PAMPA permeability of formononetin, calycosin D, onogenin, sativanone, medicarpin and maackiain were assessed for the first time in our study. The log *P* values ranged from 2.21 to 3.03 and log *D*^7.4^ values from 2.48 to 3.03, respectively, indicating optimal polarity for BBB permeation. The results of PAMPA-BBB expressed as log *P*_e_ values fell between -5.60 and -4.45, predicting their good permeation capability as well. The effective permeability values showed structure-dependent differences, indicating that the pterocarpan type skeleton was the most preferred type, followed by isoflavanones, then isoflavones. The methoxy or methylenedioxy substitution of the same skeleton did not influence the permeability significantly, contrary to an additional hydroxyl group. Membrane retention showed a similar structure dependent pattern to that of effective permeability, ranging from 16% to 70%. For the identification of volumes of chemical space related to particular biological activities the ChemGPS-NP framework was used. The MAO-B inhibitory potency and selectivity were also predicted and validated. Based on our results, MAO-B inhibitory potency could be predicted with good precision, but in the case of selectivity, only the direction could be concluded (favors MAO-B or MAO-A), not the magnitude. Our finding reflects that *Ononis* isoflavonoid aglycones show an excellent fit with the suggested parameters for BBB permeability and this is the first study to confirm the highly favorable position of these natural products for MAO-B inhibition.

## Introduction

Isoflavonoids are biologically relevant plant metabolites. The most studied group of isoflavonoids is isoflavones, the main phytochemical metabolites of e.g. soy, red clover, and kudzu root. The intensity and volume of research focusing on these plants and the isoflavones they contain is understandable, as their consumption as nutrient or dietary supplements are orders of magnitudes higher, than those of any other isoflavonoid containing plants. However, in the family Fabaceae, a wide range of herbs can be found with more exotic isoflavonoid patterns [1]. For example, in the genus *Ononis* (restharrow), beside isoflavones and isoflavanones, pterocarpans are also present [2–6]. The isoflavones of soy and red clover (genistein, daidzein, and biochanin A) are mainly regarded as phytoestrogenic compounds [7], but other isoflavonoids with various structural elements show diverse biological effects, and they have the potential to act on the central nervous system (CNS). For example, formononetin proved to be neuroprotective in traumatic brain injuries *in vivo* [8, 9], by protecting cells from oxidative stress [10, 11] and neuroinflammation [12, 13]. Formononetin also showed neuroprotective effect both *in vitro* [12, 13] and *in vivo* [14] models of Alzheimer’s disease. Other *Ononis* isoflavonoids, such as maackiain and calycosin, showed a selective MAO-B inhibiting effect *in vitro*, which was a magnitude higher than that of genistein. Lee et al. found, that maackiain is a new potent (IC_50_ = 0.68 μM), selective (selectivity index = 126.2), and reversible MAO-B inhibitor [15]. Furthermore, Oh and co-authors found similar activity for calycosin (IC_50_ = 0.24 μM, selectivity index = 293.8) and medicarpin (IC_50_ = 0.30 μM) with even more promising results [16].

Despite the promising medicinal properties of isoflavonoids, their pharmacokinetic characteristics have been described only to a limited extent, focusing mainly on the isoflavones of soy [17–20]. Generally speaking of flavonoids and isoflavonoids, it can be concluded that the absorption rates of the glycosidic forms of these compounds are very, but they are metabolized to their aglycone forms in the alimentary tract by gut microbiome [21–23]. Information is available about the oral bioavailability of medicarpin and formononetin, indicating their fast metabolization after absorption, and the low bioavailability of their aglycone form [24, 25]. On the other hand, *in vivo* studies after *per os* administration showed positive results [14, 26, 27]. The blood-brain barrier (BBB) permeability had previously been only investigated in the case of isoflavones only. These results showed, that isoflavone aglycones can permeate the BBB and that they are not P-gp substrates [20, 28, 29].

Regarding the lack of information about the BBB permeability of isoflavones, isoflavanones, and pterocarpans found in *Ononis* species, the aim of our study was to isolate these compounds in their aglycone form and investigate their physico-chemical properties influencing the absorption and distribution. In order to estimate their BBB permeability, octanol-water partitioning and the parallel artificial membrane permeability assay (PAMPA) methods were chosen, based on the known absorption characteristics of isoflavonoids—which is mainly passive, transcellular diffusion. The PAMPA was firstly introduced by Kansy et al. [30], as a cost-effective and high-throughput approach to predict the oral absorption of drugs. Later, Di and co-authors developed a PAMPA model which is suitable for the estimation of BBB permeability [31]. More recently, other research groups further optimized the assay to reach different requirements, e.g. faster incubation time or the evaluation of natural products [32–34] and applied it to investigate various natural products [35–43]. The PAMPA-BBB can be used as a screening method for plant extracts, highlighting the permeable compounds, which worth isolation [38, 39, 43], or it can be used for the assessment of BBB permeability of pure, isolated natural products and their semi-synthetic derivatives [35–37, 40, 42].

*Ononis* species contains isoflavones (calycosin D, pseudobaptigenin), isoflavanones (onogenin, sativanone), and pterocarpan (medicarpin) which are structurally similar to formononetin, calycosin, and maackiain, compounds with a probable CNS activity. The observed structural similarities raised the questions if the compounds from *Ononis* would also be active in these biological tests. To address this question, a charting of biologically relevant physico-chemical features using the ChemGPS-NP framework was employed. This framework has previously proven useful in identifying and defining volumes of chemical space related to particular biological activities [44, 45]. ChemGPS-NP also has the capacity of providing a reference system which allows the characterization and comparison of molecules of natural origin as well as those in routinely prescribed medicines [46, 47].

The aim of the study is assessing the physico-chemical properties of *Ononis* isoflavonoids influencing their permeability through the BBB for the first time by the means of octanol-water partitioning and BBB-PAMPA. Moreover, *in silico* experiments were carried out in order to investigate the permeability, the MAO-B inhibitor potency and selectivity of the target molecules.

## Materials and methods

### Solvents and chemicals

Daidzein, quinine, caffeine, naringenin, salicylic acid, and rutin standards were obtained from Sigma-Aldrich. HPLC grade acetonitrile and methanol were purchased from Merck. Ethyl-acetate, formic acid, methanol, and acetone of reagent grade were purchased from Reanal-Ker. HPLC grade water was prepared with a Millipore Direct Q5 water purification system. All aqueous eluents for HPLC were filtered through MF-Millipore membrane filters (0.45 μm, mixed cellulose esters).

### *Ononis* isoflavonoid aglycones

The isolation and identification of isoflavonoid aglycones were carried out based on the methods described in our previous papers [4, 5, 48]. The quantity and purity of the isolated substances were as follows: maackiain (12.9 mg, 100%), medicarpin (13.4mg, 100%), onogenin (12.3mg, 95%), sativanone (23.1 mg, 98%), formononetin (7.3mg, 94%), pseudobaptigenin (14.8 mg, 93%), calycosin D (4.4 mg, 92%). The purity of the isolated compounds was evaluated by UPLC and Max Plot chromatograms, see Supplementary for chromatograms. Calycosin was obtained from Sigma-Aldrich, as it could not be isolated in appropriate quantity and purity for further testing.

### Determination of log *P* values

The logarithm of octanol/water partition coefficient was measured by the validated protocol of traditional shake-flask method [49]. Both the true partition coefficient (log *P*) value and the distribution coefficient (log *D*^*pH*^) at pH 7.4 of the samples were determined. Britton-Robinson buffer (BR) served as the aqueous phase, while *n*-octanol as the organic phase. The two phases were mutually saturated with each-other. The samples were obtained in methanol. From this concentrated solution the appropriate dilution was made with the aqueous phase. Different phase ratios (R) were used for equilibration (10ml BR buffer: 0.1 mL or 0.2 mL or 0.5 mL octanol) at 25°C for 1 hour. The phases were separated by centrifugation. The decrease in concentration was determined in the aqueous phase by spectroscopy measuring the absorbance before (A_o_) and after (A_1_) the partitioning. The log *P* and log *D*^*pH*^ value was calculated using the following equation:

$$\log\;P\;\mathrm{or}\;\log\;D^{pH}=\log\;[(A_{0}-A_{1})/A_{1}]*R$$

Four parallel experiments were conducted, and average value and standard deviations (SD) were calculated. Predicted values of log *P* and p*K*_a_ were calculated using ChemAxon Marvin.

### Parallel artificial membrane permeability assay

The applied PAMPA-BBB setup was based on the work of Könczöl *et al*. [32] who validated the method for the evaluation of natural products. PAMPA “sandwiches” were formed from a Stirwell (Pion Inc.) 96-well donor and acceptor plates with a polyvinylidene difluoride filter bottom. The wells were coated with 5 μL of the solution of 16.0 mg polar porcine brain lipid (Avanti Polar Lipids Inc.) and 8.0 mg cholesterol (Sigma-Aldrich) in 600 μL of dodecane (Acros Organics). The concentrations of standard compounds and isoflavonoids of initial donor samples were approximately 10 mM in DMSO, then these samples were diluted a hundredfold with phosphate buffer saline pH 7.4 to obtain donor solutions with 1% DMSO content of which 150 μL was applied. The acceptor compartment was filled with 180 μL phosphate buffer saline pH 7.4. The sandwiches were incubated in a water vapor-saturated atmosphere at 37°C for 4 hours in the Gut-Box (Pion Inc.) module with stirring to adjust the thickness of the aqueous boundary layer to 60 μm. Three to six parallel measurements were made for each sample and the assays were repeated three consecutive days. Sample concentrations in the acceptor and donor wells were determined by HPLC [33]. For this, a Waters Acquity UPLC system was furnished with a sample manager, a binary solvent manager, and a PDA detector (Waters Corporation). The samples were subsequently analyzed on the Acquity UPLC with a DEH C18 column (2.1 x 100 mm, 1.7 μm) using eluents 0.1% formic acid (A) and acetonitrile (B) at 0.3 mL/min flowrate. To determine the standard compounds, the following gradient program was used: 0 min 5% B, 5 min 100% B.

For the quantification of isoflavonoids an isocratic system was used with 40% B. Retention (capacity) factors were calculated from the chromatographic runs detailed above, as follows:

$$k=\frac{t_{R}-t_{0}}{t_{0}}$$ [1]

The effective permeability and the membrane retention of the compounds were calculated by the following equations [50]:

$$Pe=\frac{-2.303}{A(t-\tau_{ss})}*\left(\frac{V_{A}*V_{D}}{V_{A}+V_{D}}\right)*lg\left[1-(\frac{V_{A}+V_{D}}{(1-MR)*V_{D}})*\frac{C_{A}(t)}{C_{D}(0)}\right]$$ [2]

where *Pe* is the effective permeability coefficient (cm/s), *A* is the filter area (0.24 cm^2^), *V*_*D*_ and *V*_*A*_ are the volumes in the donor (0.15 cm^3^) and acceptor phases (0.18 cm^3^), *t* is the incubation time (s), *τ*_*SS*_ is the time (s) to reach steady-state (240 s), *C*_*A*_*(t)* is the concentration (mol/cm^3^) of the compound in the donor phase at time *t*, *C*_*D*_*(0)* is the concentration (mol/cm^3^) of the compound in the donor phase at time 0, *C*_*D*_*(t)* is the concentration (mol/cm^3^) of the compound in the donor phase at time *t*, and *MR* is the estimated membrane retention factor.


$$MR=1-\frac{C_{D}(t)}{C_{D}(0)}-\frac{V_{A}}{V_{D}}*\frac{C_{A}(t)}{C_{D}(0)}$$ [3]

Prior to analyzing isoflavonoid aglycones, standard compounds were applied (daidzein, quinine, caffeine, naringenin, salicylic acid, and rutin) to verify the system suitability (see Supplementary material).

### Cheminformatics

Chemical space analysis was performed using the principal component analysis (PCA)-based chemical space navigation tool ChemGPS-NP [45, 47] which is freely available online at http://www. chemgps.bmc.uu.se/. The reference sets of BBB passive diffusers, non-diffusers and P-glycoprotein substrates were based on the thesis of L. Viklund [51] titled ‘ChemGPS-NP as a tool for predicting drug distribution across the blood-brain barrier’. Monoamine oxidase A and B inhibitors were collected from the ChEMBl database searching for the human enzymes and using the cut-off value of IC_50_ < 10 μM. For evaluating activity, all MAO-B inhibitors were used over the cutoff value. For evaluating the selectivity, only molecules with a known MAO-A and B inhibitor activity were used. Selectivity index was calculated based on the IC_50_ values of the reference structures (IC_50_ MAO-B/IC_50_ MAO-A). Euclidean distances were calculated between points P = (p1, p2,…, p8) and Q = (q1, q2,…, q8) in Euclidean 8D space provided by the ChemGPS-NP coordinates using the following equation in Excel:

$$Euclidean\;distance=\sqrt{{\left(p_{1}-q_{1}\right)}^{2}+{\left(p_{2}-q_{2}\right)}^{2}+\cdots +{\left(p_{8}-q_{8}\right)}^{2}}$$ [4]

The first three dimensions (plotted in Plotly Chart studio) of the ChemGPS-NP map, can be interpreted in such a way that the first dimension (principal component one, PC1) represents size, shape and polarizability; PC2 corresponds to aromatic and conjugation-related properties; PC3 describes lipophilicity, polarity, and H-bond capacity. For the purpose of visualization, only the first three dimensions were used but the calculations were based on all eight dimensions.

## Results and discussion

### Lipophilicity characterized by the log D^pH^ / log P values

**Fig 1:**
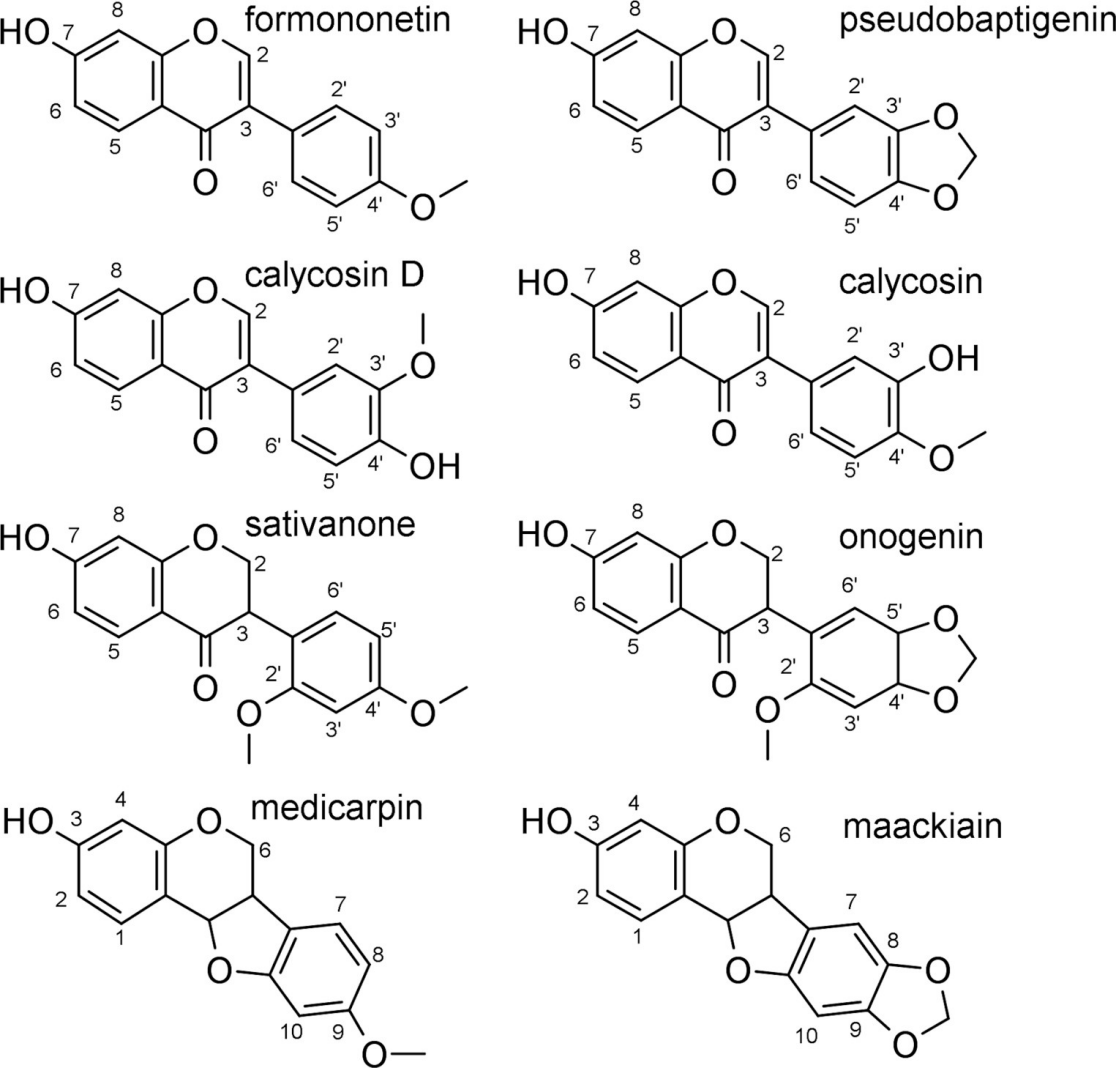
The structure and numbering of the investigated isoflavonoid aglycones.

The lipophilicity of drugs is an essential feature determining their route in living systems, including their absorption, distribution, accumulation, and elimination. The octanol/water partition coefficient is a generally accepted physico-chemical parameter for the characterization of lipophilicity. The preliminary p*K*_a_ predictions suggested that methoxylated isoflavones have a p*K*_a_ value around 7, which was confirmed by literature sources [52, 53]. Because of the methylation in the 4’-OH position, the 7-OH group attached to the chromenone moiety has a p*K*_a_ value lower than expected (Fig 1).

**Fig 2:**
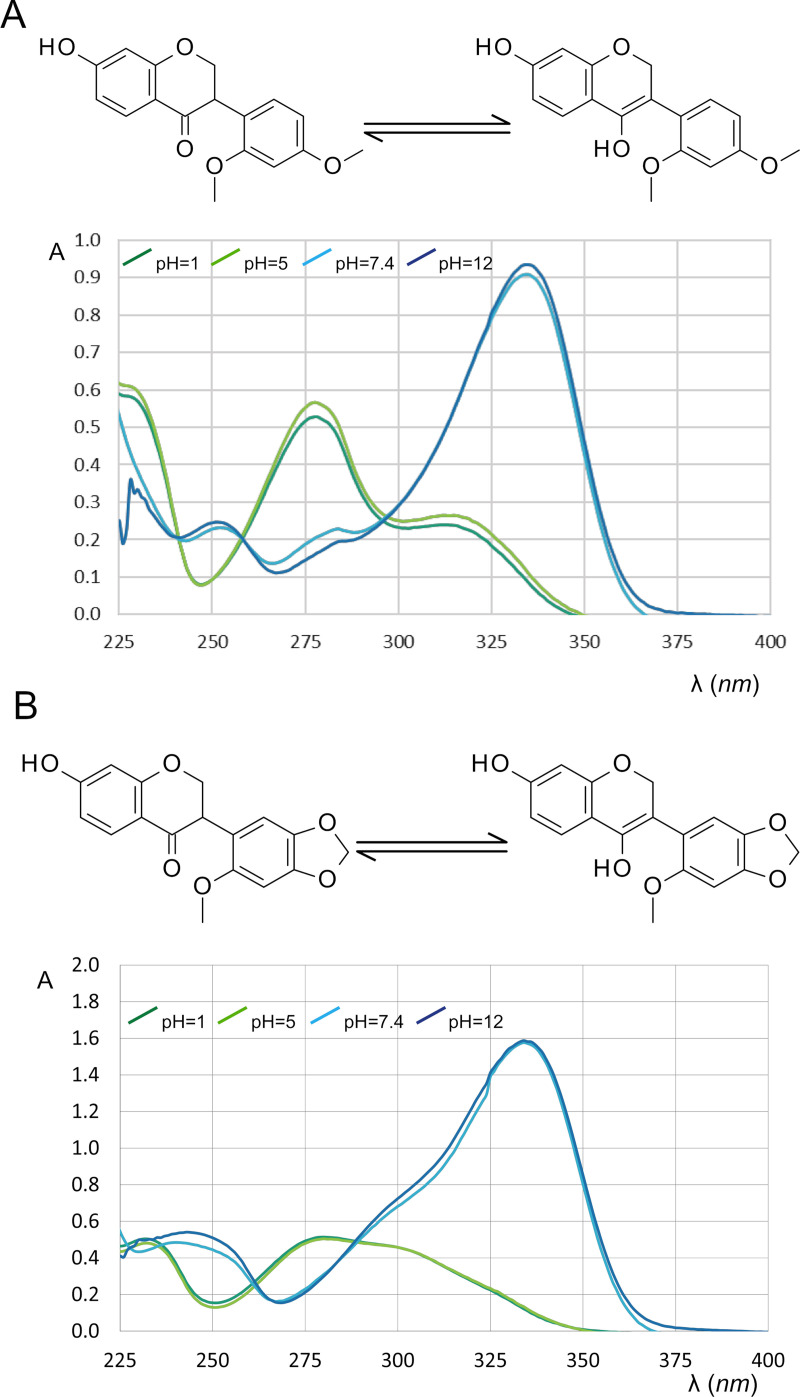
The oxo-enol tautomerism and pH dependent UV spectra of sativanone (A) and onogenin (B).

Therefore, the log *P* value of isoflavone derivatives (formononetin and calycosin) was determined at pH 5. In the case of isoflavanones, the calculations predicted a p*K*_a_ of 7.78 which could not be explained by the extensive conjugated electron system of the chromenone ring as the bond between C2 and C3 is saturated (see Fig 1). On the other hand, these molecules have the ability of oxo-enol tautomerism [54] (Fig 2), which could amplify the acidic character of the 7-OH group, so their log *P* value was determined based on the partition at pH 5.

Fig 2A and 2B show the pH dependent UV spectra of these two molecules. Based on these data we concluded that the two molecules are fully unionized at pH 5. Regarding pterocarpans, ring A, which bears the ionizable 3-OH group (Fig 1), is not linked to other parts of the molecule with conjugated bonds, and thus the calculated p*K*_a_ value is close to that of a phenolic hydroxyl group.

In our results (Table 1), all compounds showed favorable log *P* and log *D*^*7*.*4*^ values regarding drug-likeness and Lipinski’s rule of five, indicating, that the studied compounds are able to passively permeate through biological membranes. Those structures which have lower p*K*_a_ values (calycosin D, formononetin, onogenin and sativanone) showed somewhat higher log *D*^*pH*^ values at pH 5 than at pH 7.4. The difference between the log *D*^*pH*^ values was higher in the case of isoflavanones, whereas for formononetin, the difference is measurable but not significant. The highest log *D*^*7*.*4*^ value was measured for medicarpin, which were followed by sativanone and formononetin, then onogenin and maackiain. The least lipophilic compound is calycosin D, with an order of magnitude lower lipophilicity than that of medicarpin (Table 1).

**Table 1:** The calculated p*K*_a_ values, experimental log *D*^*pH*^ and log *P* values and wavelength used during the measurements of the six isoflavonoids.

Name	Calculated p*K*_a_	Measured log *D*^*5*^ ± SE	Measured log *D*^*7*.*4*^ ± SE	Experimental log *P*	Absorbance maximum
**Calycosin D**	6.48, 9.21	2.48 ± 0.02	2.21 ± 0.04	2.48	255 nm
**Formononetin**	6.48	2.83 ± 0.05	2.73 ± 0.07	2.83	251 nm
**Sativanone**	7.78	3.04 ± 0.01	2.75 ± 0.03	3.04	277 nm
**Onogenin**	7.78	3.03 ± 0.02	2.49 ± 0.04	3.03	280 nm
**Medicarpin**	9.34	-	3.03 ± 0.02	3.03	284 nm
**Maackiain**	9.34	-	2.49 ± 0.02	2.49	311 nm

### In vitro PAMPA-BBB permeability

In this study, a PAMPA-BBB method, previously validated for natural compounds was utilized [32] with a slight modification using stirring in wells with an unstirred water layer of 60 μm. In the first step, we used standard molecules (daidzein, quinine, caffeine, naringenin, salicylic acid, and rutin) for system validation. In our results, the measured log *P*_e_ values showed good correlation (R^2^ = 0.9498) with the log *BB* values from literature (see S1 Table and S1 Fig in S1 File), verifying the appropriate predictive power of the system.

Molecules with very high or very low log BB values on the scale could be easily defined as well permeating (BBB+) or not sufficiently permeating (BBB-). As log *BB* and CNS permeability are continuous data, it is hard to form only two categories, and to characterize a single value which demarcates BBB+ and BBB- molecules. In the work of Mensch *et al*. the condition for BBB+ category is to have a log *BB* value ≥ 0 and they determined the *in vitro* limit differently, based on the specificities of the applied PAMPA-BBB test [34]. Könczöl *et al*. regarded molecules as BBB+ above a log *BB* -0.5 value and defined the arbitrary unit of -6.0 of log *P*_e_ as an effective discriminator between BBB+ and BBB- structures. Di *et al*. determined an uncertain, intermediate zone between *P*_e_ = 2 x 10^−6^ cm/s and *P*_e_ = 4 x 10^−6^ cm/s (log *P*_e_ -5.69 to -5.39) [31, 32].

**Fig 3:**
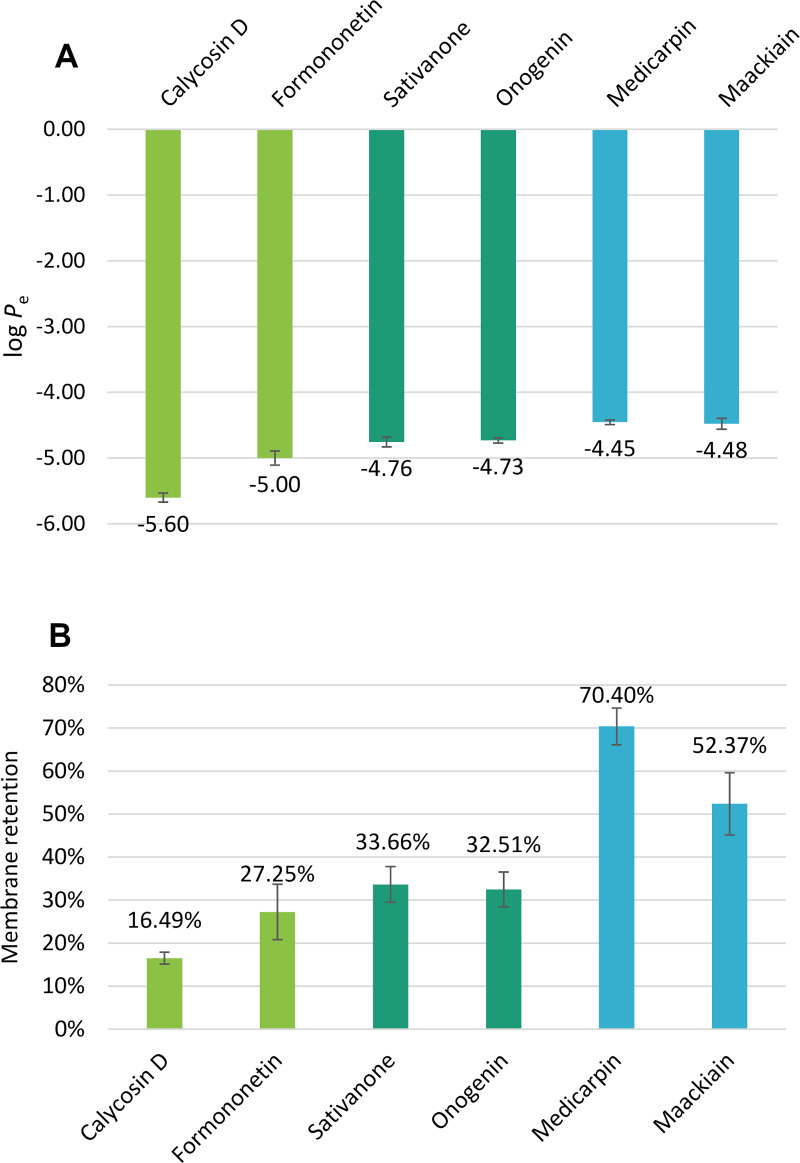
Experimental log *P*_e_ values (A) and membrane retention (B) of the six isoflavonoid compounds (bright green: isoflavones, deep green: isoflavanones, bright blue: pterocarpans).

After analyzing the experimental log *P*_e_ values (S2 Table in S1 File), structural correlation can be observed. The isoflavones calycosin D and formononetin had the lowest log *P*_e_ values, followed by isoflavanones sativanone and onogenin, while the highest values were measured for pterocarpans maackiain and medicarpin. The methoxy or methylenedioxy substitution of the same skeleton caused no significant differences in log *P*_e_ values (onogenin vs. sativanone, medicarpin vs. maackiain), but an additional hydroxyl group contributed significantly (formononetin vs. calycosin D) (Figs 1 and 3A). Regarding the different literature cut off values in distinction of BBB+ and BBB- structures found in literature, all compounds, except calycosin D, fell into the well-permeating category, therefore they can be considered permeating through the blood-brain barrier via passive diffusion. Calycosin D could be considered as well-permeating based on the works of Könczöl *et al*. However, according to Mensch *et al*., it would be categorized as BBB-, while using the categorization of Di *et al*. it could be classified as uncertain with a *P*_e_ value of 2.5 x 10^−6^ cm/s [31, 32, 34]. Taking into consideration, that the structural isomer calycosin, which only differs slightly in the position of a methyl group, has been reported by several studies as a neuroprotective agent [55–57], it can be assumed that calycosin D can reach the cells of the brain. Comparing the permeability of structures studied, more than one order of magnitude difference could be drawn between the most and least permeable compounds (medicarpin and calycosin D, respectively). The membrane retention showed a somewhat similar pattern, with an average value of 20% for isoflavones, 33% for isoflavanones and 61% for pterocarpans, but the distinction between isoflavonoid skeletons is less pronounced (Fig 3B). Medicarpin showed the highest membrane retention, which can be correlated with its highest log *D*^7.4^ value (see Table 1).

Table 2 highlights an almost perfect fit between the isoflavonoids and the suggested BBB permeability [58]. Only calycosin D shows more than one outlier, and the polar surface area of onogenin is higher than recommended. Based on these results, the physico-chemical parameters of *Ononis* isoflavonoids are appropriate for further drug development aiming the CNS.

**Table 2:** Suggested physicochemical property ranges for increasing the potential for BBB penetration and physicochemical features of the six isoflavonoid aglycones.

Compound	Molecular weight (Da)	PSA (Å^2^)	HBD	log *P*	log *D*^7.4^	log *P*_e_
**Preferred range**	<450	<70	0–1	2–4	2–4	>-5.50
**Calycosin D**	284.27	75.99	2	2.48	2.21	-5.60
**Formononetin**	268.27	55.76	1	2.83	2.73	-5.00
**Sativanone**	300.31	64.99	1	3.04	2.75	-4.76
**Onogenin**	314.29	74.22	1	3.03	2.49	-4.73
**Medicarpin**	270.25	47.92	1	3.03	3.03	-4.45
**Maackiain**	284.27	57.15	1	2.49	2.49	-4.48

PSA: Polar Surface Area, HBD: Number of hydrogen-bond donors.

### Cheminformatics

#### Isoflavonoid BBB permeability

**Fig 4:**
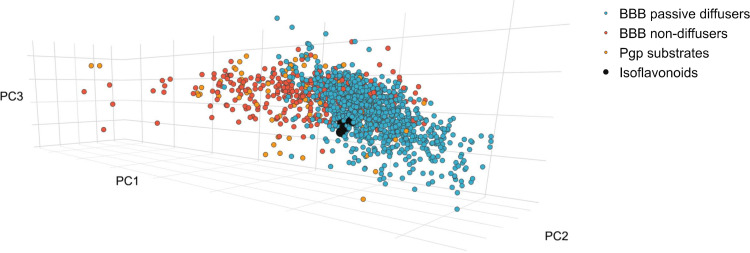
The location of BBB passive diffusers (blue), non-diffusers (red), P-glycoprotein substrates (orange) and isoflavonoids (black) in chemical space.

In order to corroborate the *in vitro* results of isoflavonoids permeating the BBB, we investigated the location of the structures studied in the chemical space in the proximity of known passive diffusers and non-diffusers. Based on the plot, the isoflavonoids merge to the cluster of BBB passive diffusers and do not have common chemical space with non-diffuser molecules, confirming the transcellular passive diffusing mechanism of BBB permeability and the accuracy of the PAMPA studies (Fig 4). Most submerged in the cloud of passive diffusers are onogenin and sativanone can be observed, with relatively the highest PC1 and lowest PC2 values, which can be the result of the saturated ring C (lower aromaticity) together with the high number of *O* atoms (higher polarizability) (see Fig 1). In Fig 4 it can be seen that the substrates of P-gp are not clustered together with *Ononis* isoflavonoids, but rather with non-diffuser molecules.

#### Isoflavonoids and monoamine oxidases

**Fig 5:**
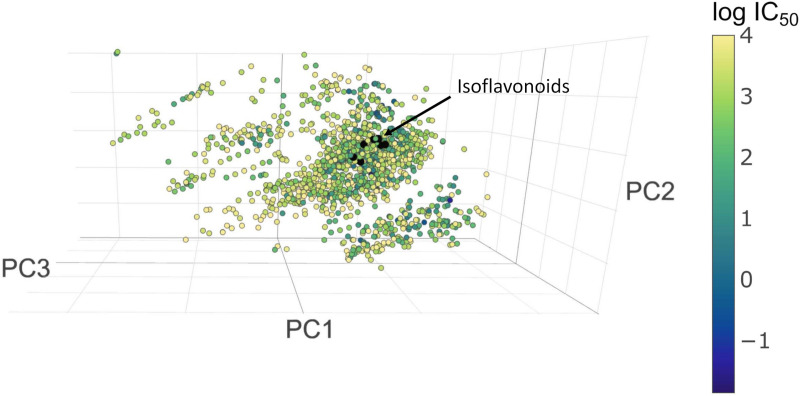
The location of MAO-B inhibitors and *Ononis* isoflavonoids in chemical space (blue color correlates with lower IC_50_ values, yellow with higher ones).

MAO inhibitors are mainly used in psychiatry for the treatment of depressive disorders, and anxiety disorders and in neurology for the treatment of Parkinson’s disease and Alzheimer’s disease. While the classical non-selective and irreversible MAO inhibitors are characterized by the risk of inducing a hypertensive crisis, the selective MAO-B inhibitor selegiline and the selective and reversible inhibitor of MAO-A, moclobemide, are free from this potential interaction [59]. This emphasizes the significance of the previous results, where the selected isoflavonoids seemed to be selective and reversible inhibitors of MAO-B [15, 16]. Having evaluated the first dataset describing the position of MAO-B inhibitors with IC_50_<1000 nM it can be observed that *Ononis* isoflavonoids can be found in the same cluster as MAO-B inhibitors (Fig 5).

As compounds with lower than 1 EDs are considered to be close in the chemical space [44], firstly their number were assessed around the target compounds. Calycosin and calycosin D could be regarded as identical in this system, as their ED was 0. Formononetin showed the highest number of MAO-B inhibitors in its proximity, whereas maackiain had no close neighbors. In the next step, the IC_50_ values of the closest 10 and 5 molecules were investigated and compared with *in vitro* results from literature [15, 16]. Surprisingly, maackiain with no other compounds in tight closeness, had a group of surrounding molecules with one of the most potent characteristics. The estimated IC_50_ values (0.41μM and 0.51 μM) fell very close to its *in vitro* value (0.68 μM) [15]. In the case of medicarpin, the estimation (0.60 μM and 0.21 μM) proved to be appropriate (*in vitro* 0.30 μM) [16]. Calycosin and calycosin D showed the highest estimated average IC_50_ value (1.9 and 2.1 μM) which does not correlate with the experimental data (0.24 μM). On the other hand, the compounds around calycosin showed a high deviancy in their IC_50_ values (45 nM– 8710 nM). Additionally, calycosin had the lowest Euclidean distance (0.06) to a standard molecule, acacetin, which has an outstandingly low IC_50_ value (49 nM). Other isoflavonoid compounds which had not been tested before (formononetin, onogenin, sativanone, pseudobaptigenin), showed very promising estimated results, too (see Table 3).

**Table 3:** The number of compounds in the proximity of the target molecules, and the average MAO-B inhibitory IC_50_ values of the 10 and 5 closest structures in chemical space (see details in supplementary).

	No of Compounds (ED<1)	Average ED		Average IC_50_ (nM)	
		5 closest	10 closest	5 closest	10 closest
**Formononetin**	184	0.41	0.48	605	515
**Calycosin/Calycosin D**	31	0.48	0.60	2092	1930
**Pseudobaptigenin**	32	0.82	0.86	1390	1750
**Sativanone**	59	0.53	0.55	499	495
**Onogenin**	27	0.74	0.78	632	996
**Medicarpin**	43	0.52	0.63	210	604
**Maackiain**	0	1.17	1.19	513	407

Comparing the position of isoflavonoids and reference compounds with increased activity, they showed a higher value in PC3 indicating more lipophile compounds. This trend can be observed regarding the experimental log *P* values and the estimated activity of our molecules, too. As a consequence, increasing the apolar character of the tested molecules may increase the enzyme inhibitory activity.

**Fig 6:**
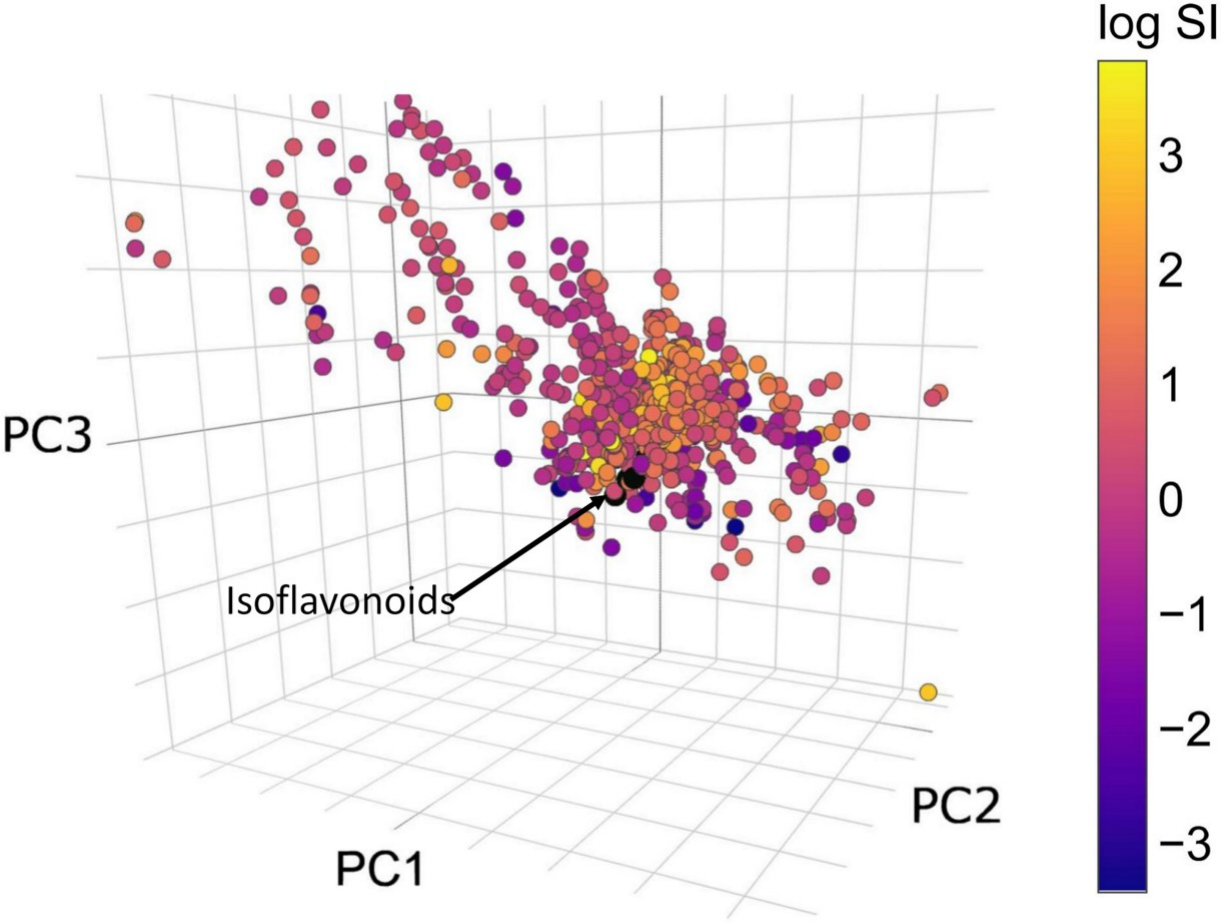
The location of MAO-A and MAO-B inhibitors beside *Ononis* isoflavonoids in chemical space color coded by their selectivity index (purple: Inhibiting selectively MAO-A, yellow: Inhibiting selectively MAO-B).

In the next step, molecules from the first dataset were selected with known MAO-A inhibitory effect, then the selectivity index was calculated and plotted in chemical space (see Fig 6). Purple color shows a higher selectivity for MAO-A, while yellow is the indicator of selective MAO-B inhibition.

The same calculations were carried out, as previously, the results can be found in Table 4. The number of compounds with ED<1 is lower for each molecule, since fewer compounds were tested. Altogether, all isoflavonoids showed an estimated preference for the MAO-B enzyme, but the predicted selectivity indices are an order of magnitude lower, than the experimental ones (for maackiain, calycosin and medicarpin). These results both emphasize the strength and the limitation of the ChemGPS-NP framework, as the trends of the selectivity could be predicted, however, the exact values not. Onogenin and sativanone seems to be promising candidates for further testing based on their estimated values. More selective molecules have higher PC3 values, indicating the possible beneficial effect of increasing the lipophilicity of the tested molecules again.

**Table 4:** The number of compounds in the proximity of the target molecules, and the average MAO-B inhibitory selectivity indices of the 10 and 5 closest structures in chemical space (see details in supplementary).

	No of Compounds (ED<1)	Average ED		Average Selectivity Index	
		5 closest	10 closest	5 closest	10 closest
**Formononetin**	70	0.64	0.68	8.87	6.97
**Calycosin/Calycosin D**	14	0.50	0.65	7.28	11.54
**Pseudobaptigenin**	13	0.82	0.88	22.32	12.00
**Sativanone**	17	0.74	0.81	55.01	28.58
**Onogenin**	8	0.83	0.92	8.12	29.18
**Medicarpin**	20	0.69	0.72	11.10	30.53
**Maackiain**	0	1.16	1.22	10.93	24.93

## Conclusion

In this work, the physico-chemical properties such as lipophilicity (expressed with the logarithm of octanol-water partition coefficient) and permeability (measured by BBB-PAMPA assay) of formononetin, calycosin D, onogenin, sativanone, medicarpin and maackiain were characterized for the first time. As a result, optimal log *P* and log *D*^7.4^ values were found for passive diffusion through the BBB. Before investigating isoflavonoid aglycones, the PAMPA-BBB system was verified using standard molecules. The obtained effective permeability values of *Ononis* isoflavonoids indicated excellent permeability. Structure—log *P*_e_ relationships were deduced, as major differences were found for the various aglycones, but their methoxylation or methylenedioxy-substitution showed insignificant differences. Although pterocarpan derivatives showed the most preferable log *P*_e_ values, their membrane retention was the highest, too. Plotting the target isoflavonoids in the chemical space together with passive diffusers, P-gp substrates and non-diffusers using the ChemGPS-NP framework confirmed the hypothesized transcellular passive diffusion as a route of absorption and distribution. Investigating the closest known MAO-B inhibitors in chemical space, the predicted IC_50_ values showed good correlation with *in vitro* values. When comparing the selectivity of the predicted values with the experimental ones, only the preference could be estimated, but values could not. Considering our results, formononetin, onogenin, pseudobaptigenin and sativanone are predicted inhibitors of MAO-B, making them good candidates for future *in vitro* and *in vivo* tests.

## Supporting information

S1 File (DOCX)

S2 File (XLSX)

S3 File (XLSX)

S4 File (ZIP)
