# Supplementary material for: In vitro and in silico evaluation of Ononis isoflavonoids as molecules targeting the central nervous system
Source: PLoS One. 2022 Mar 17;17(3):e0265639. doi: 10.1371/journal.pone.0265639 (PMC8929578; doi:10.1371/journal.pone.0265639)
Supplement: S1 File — (DOCX) [file pone.0265639.s001.docx]

Title: *In vitro* and *in silico* evaluation of *Ononis* isoflavonoids as molecules targeting the central nervous system

Nóra Gampe^1^, Dominika Noémi Dávid^1^, Krisztina Takács-Novák^2^, Anders Backlund^3^, Szabolcs Béni^a1^*

^1^Department of Pharmacognosy, Semmelweis University, Budapest, Hungary

^2^Department of Pharmaceutical Chemistry, Semmelweis University, Budapest, Hungary

^3^Department of Pharmaceutical Biosciences, Pharmacognosy, Uppsala University, Uppsala, Sweden

*Corresponding author:

Email addresses: [beni.szabolcs@pharma.semmelweis-univ.hu](mailto:beni.szabolcs@pharma.semmelweis-univ.hu) (SB)

Table S1: Permeability data of standard compounds used for validation of PAMPA-BBB assay

| Standard compound | log *BB* literature value | Reference | log *P*_e_ experimental value (n=6) |
| --- | --- | --- | --- |
| Quinine | 0.60 | (Mensch et al., 2010) | -4.81 ± 0.05 |
| Salicylic acid | -1.10 | (Mensch et al., 2010) | -8.07 ± 0.01 |
| Atropine | -0.06 | (Mensch et al., 2010) | -5.35 ± 0.04 |
| Caffeine | 0.01 | (Di et al., 2003; Könczöl et al., 2013) | -5.49 ± 0.01 |
| Daidzein | -0.15 | (Mensch et al., 2010) | -5.47 ± 0.03 |
| Rutin | -1.22 | (Di et al., 2003; Könczöl et al., 2013) | -8.11 ± 0.01 |


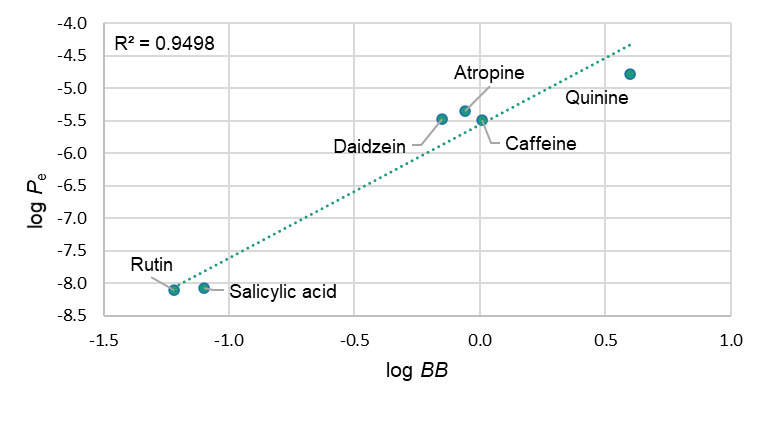


Fig S1: The *in vivo* – *in vitro* correlation between experimental log *BB* values and effective permeabilities determined by PAMPA-BBB


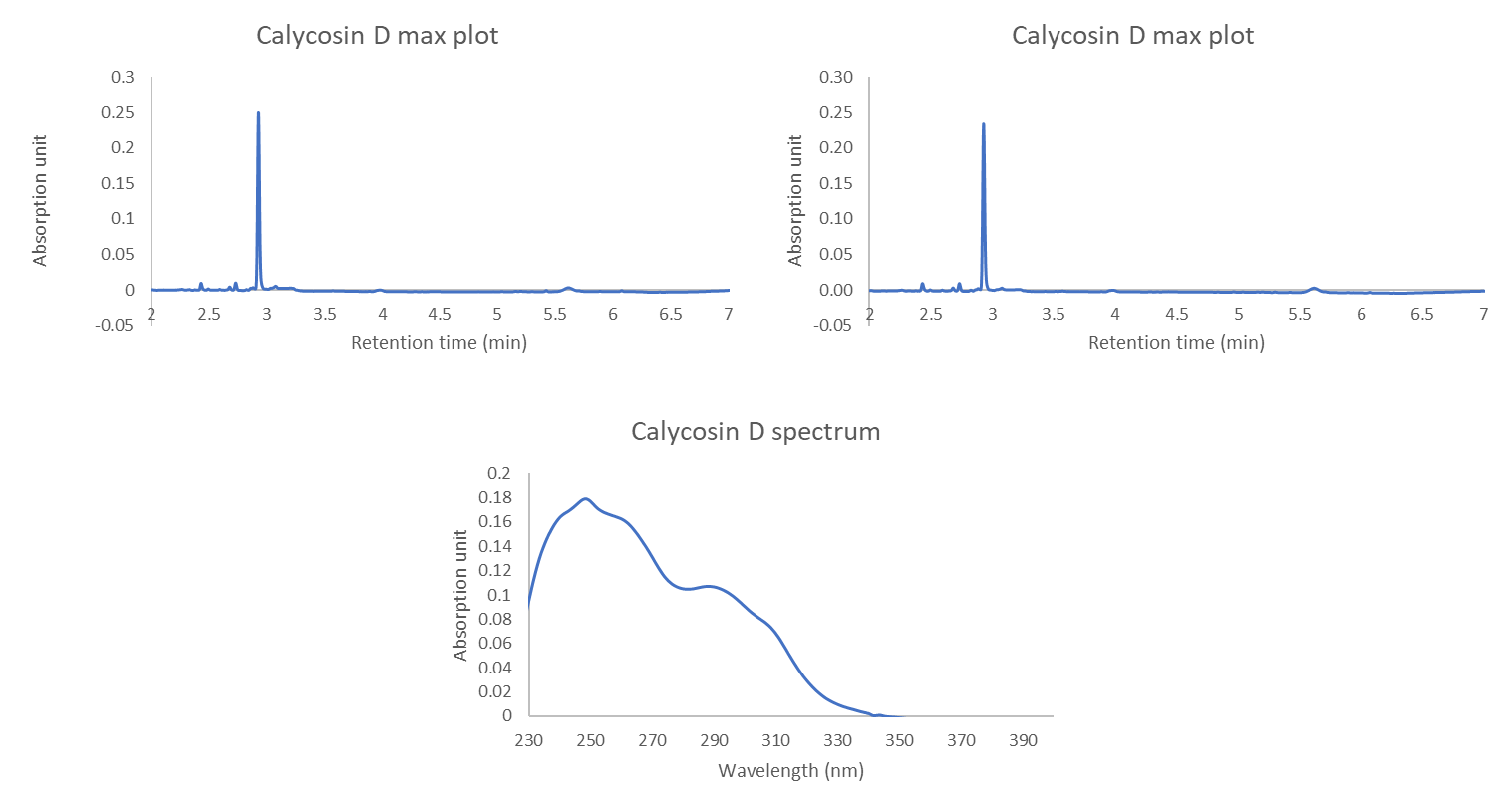


Fig S2: UPLC chromatogram and spectrum of isolated calycosin D


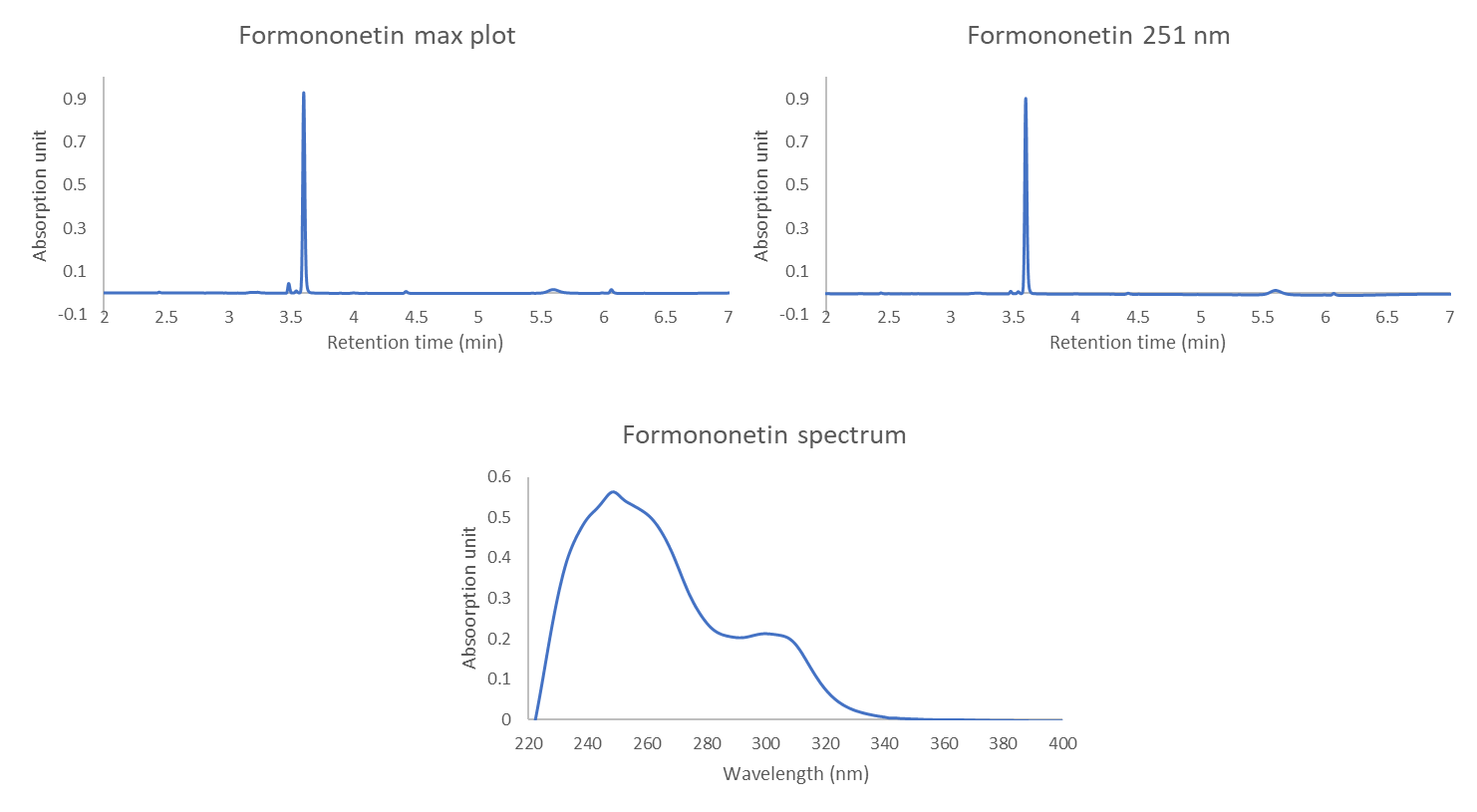


Fig S3: UPLC chromatograms and spectrum of isolated formononetin


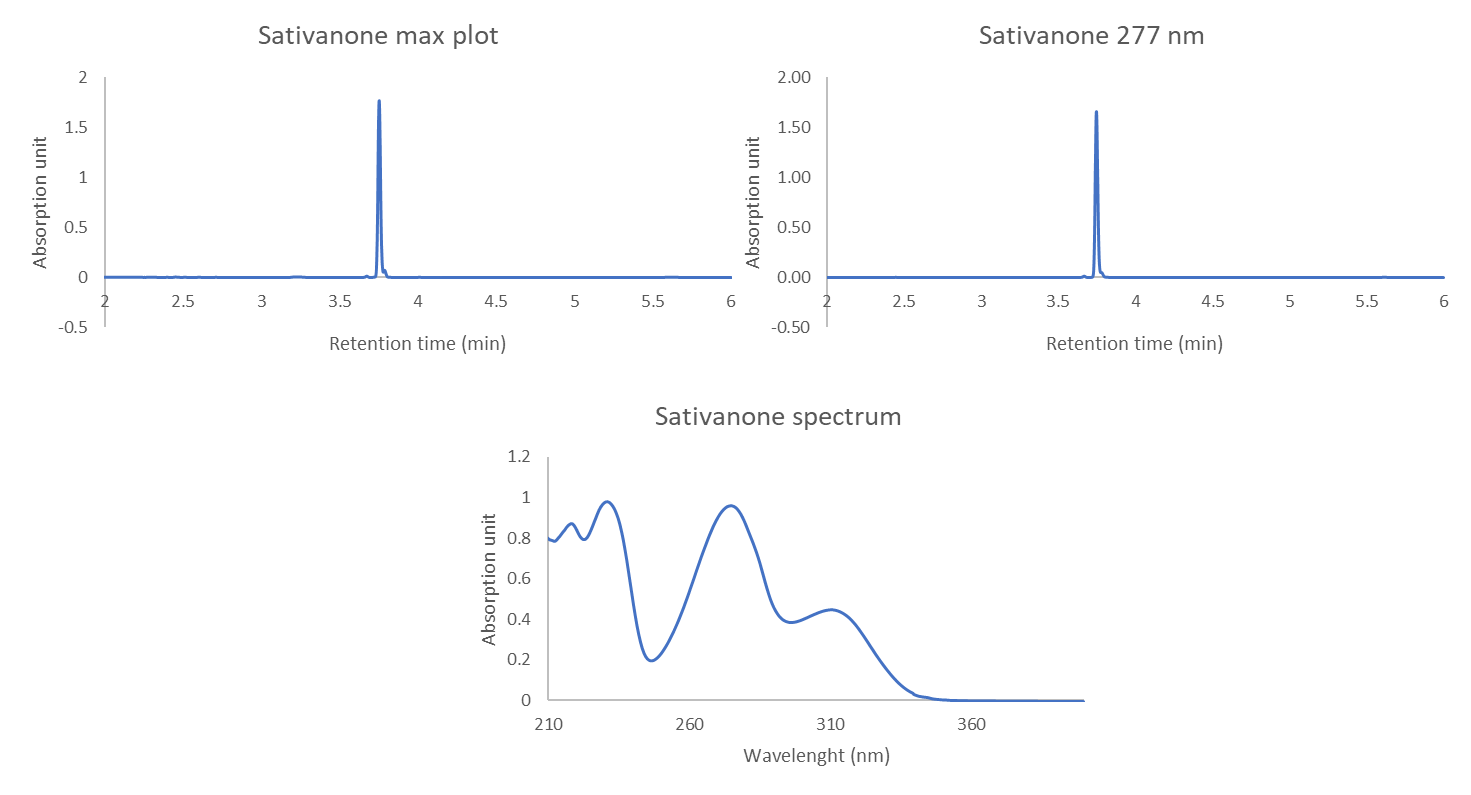


Fig S4: UPLC chromatograms and spectrum of isolated sativanone


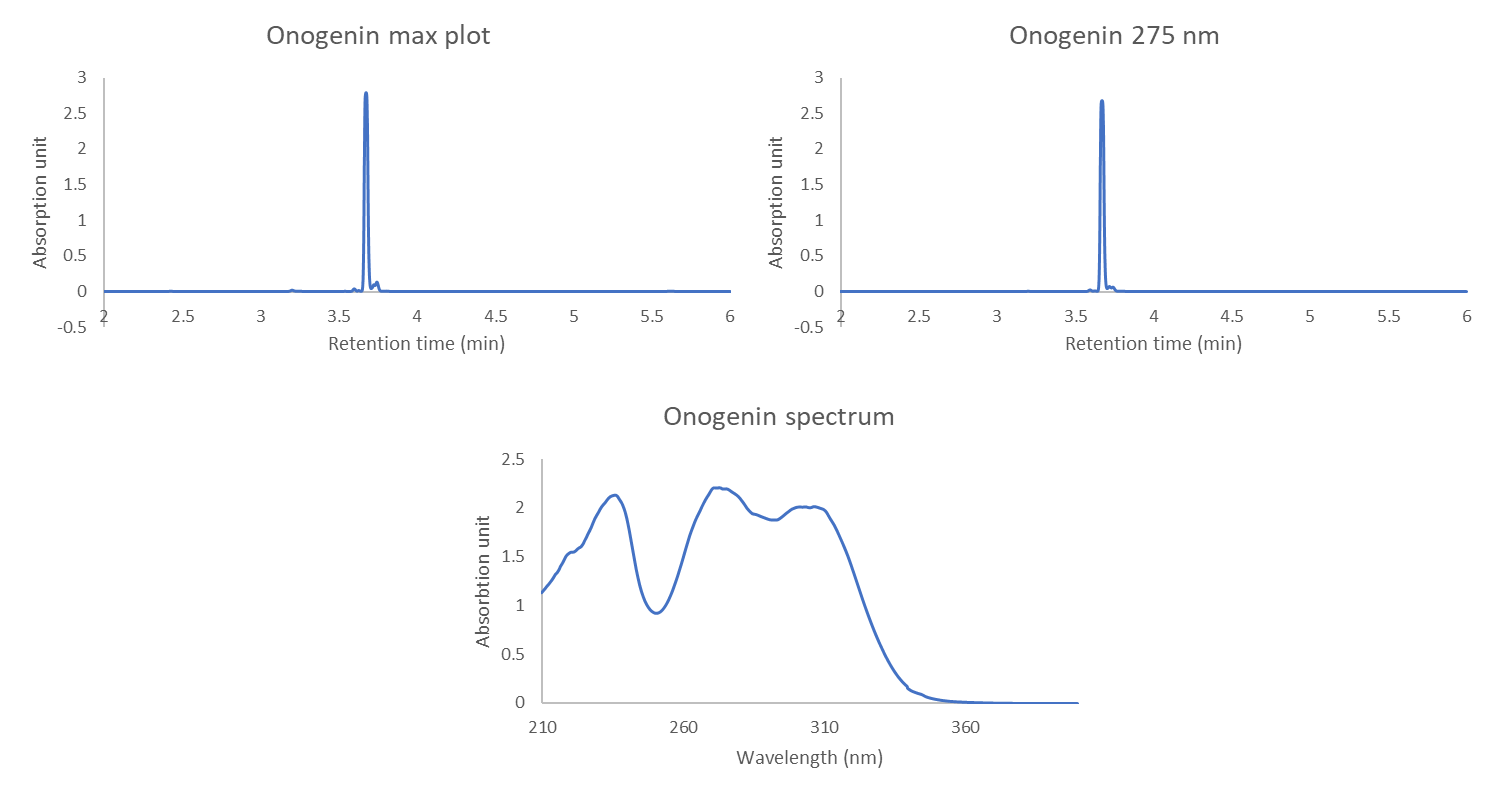


Fig S5: UPLC chromatograms and spectrum of isolated onogenin


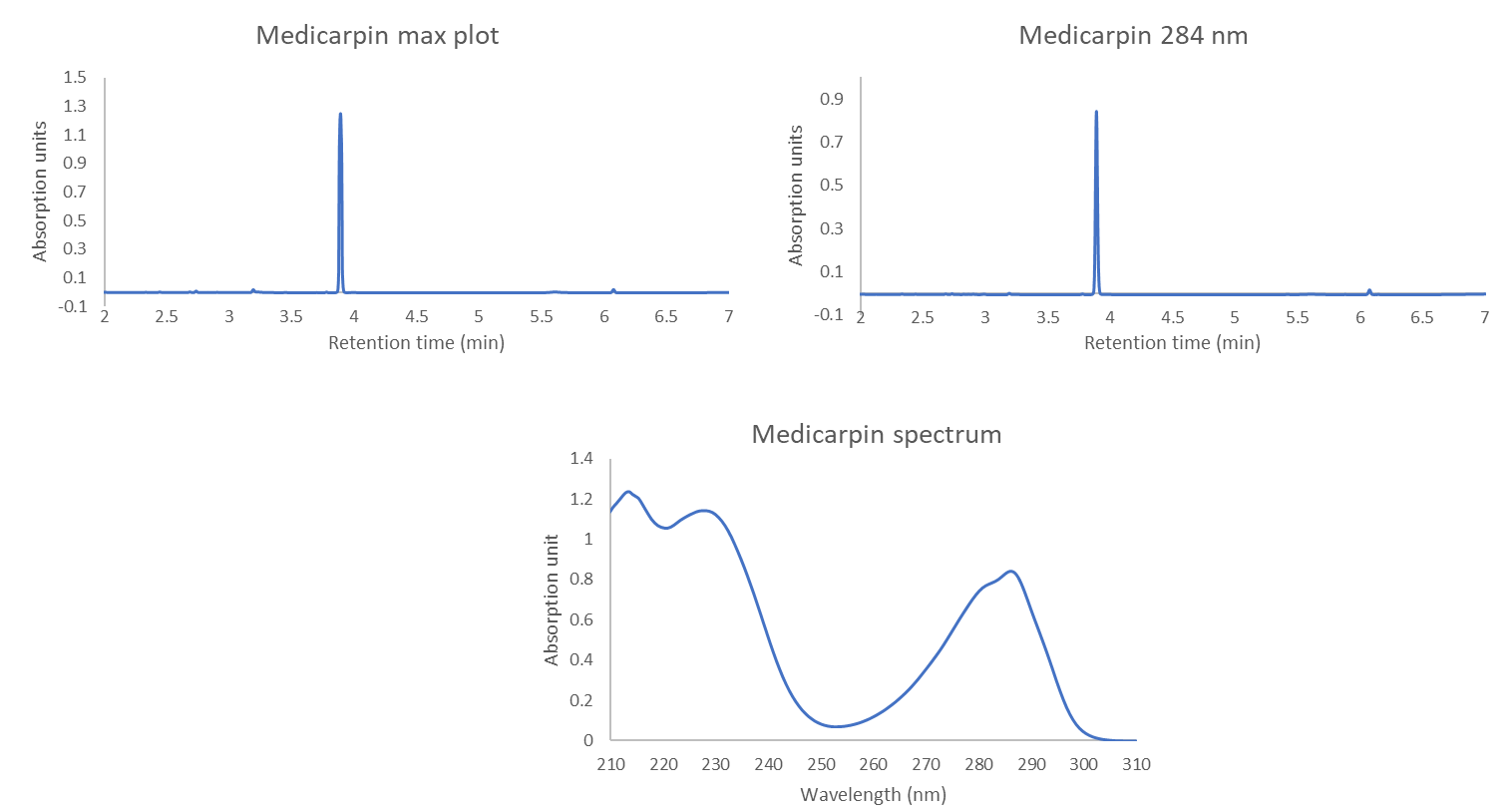


Fig S6: UPLC chromatograms and spectrum of isolated medicarpin


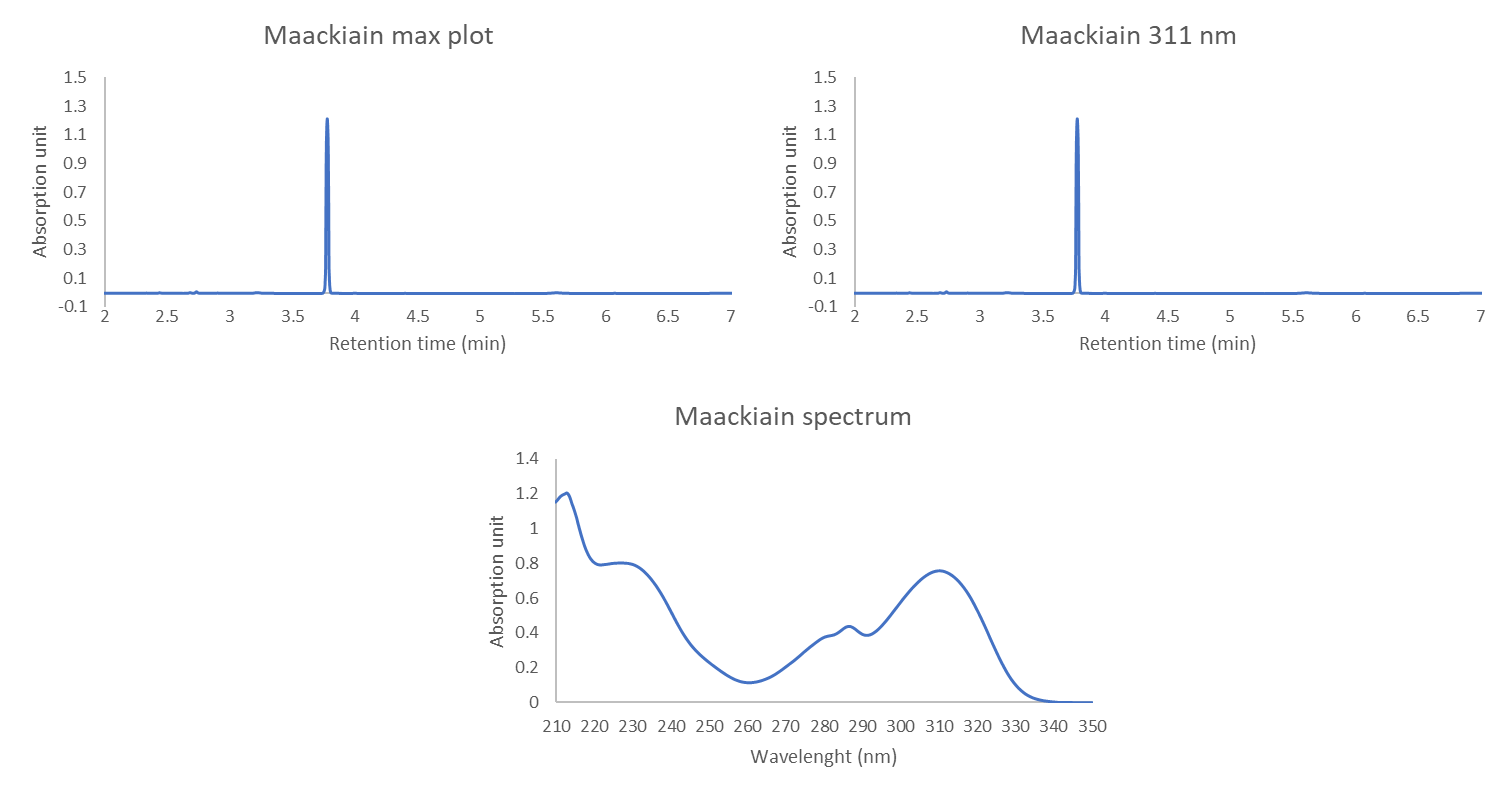


Fig S7: UPLC chromatograms and spectrum of isolated maackiain

Table S2: Experimental log *P*_e_ values and membrane retention of isolated *Ononis* isoflavonoids

| Compound | Experimental log *P*_e_ value ± SD (n=9) | Membrane retention ± SD  (n=9) |
| --- | --- | --- |
| Calycosin D | -5.60 ± 0.07 | 16.49 ± 1.38% |
| Formononetin | -5.00 ± 0.11 | 27.25 ± 6.43% |
| Sativanone | -4.76 ± 0.07 | 33.66 ± 4.13% |
| Onogenin | -4.73 ± 0.04 | 31.51 ± 4.04% |
| Medicarpin | -4.45 ± 0.04 | 70.40 ± 4.25% |
| Maackiain | -4.48 ± 0.08 | 52.37 ± 7.25% |


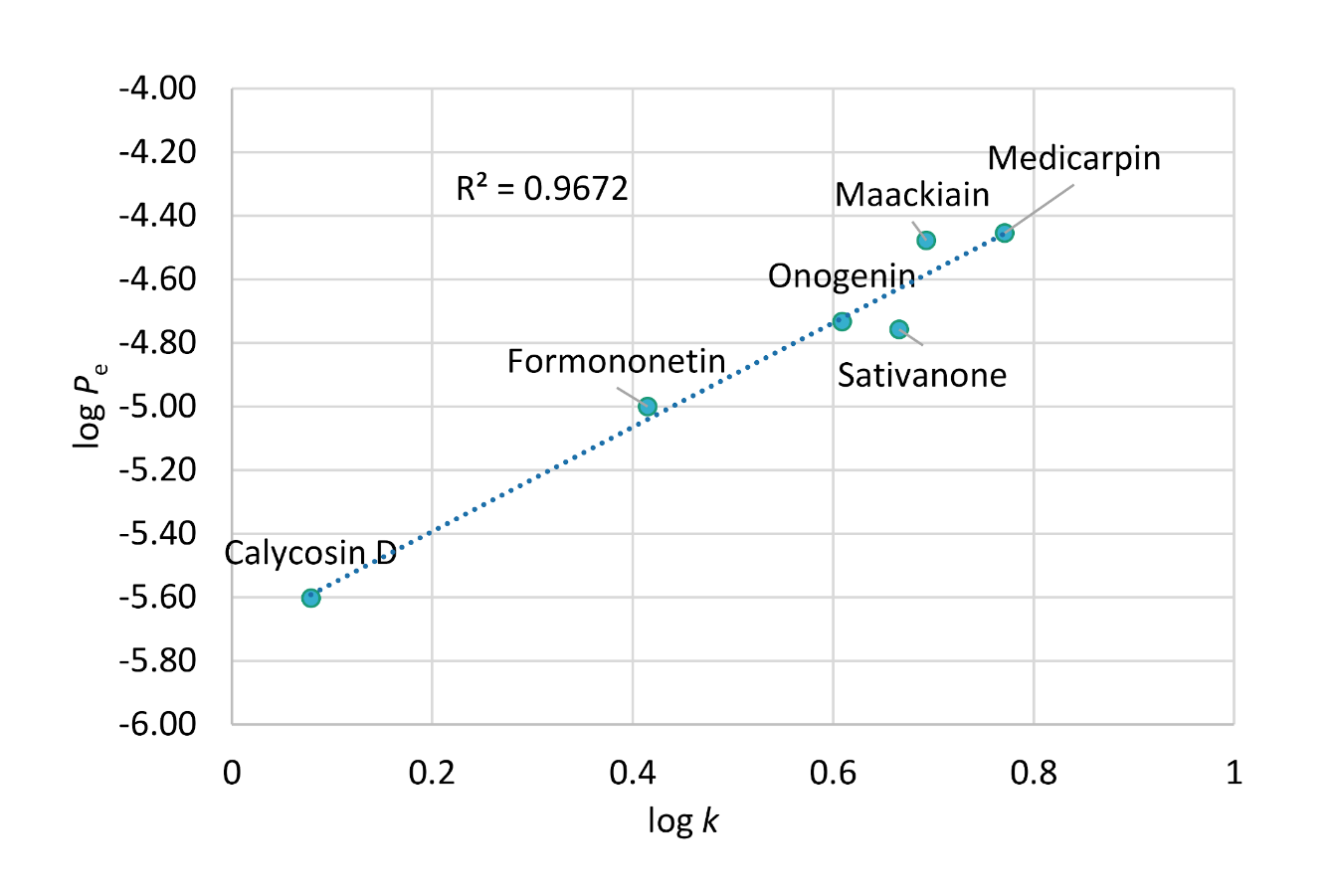


Fig S8: The correlation between the logarithm of the retention (capacity) factor (log *k*) and the logarithm of effective permeability (log *P*_e_)

Comparing the octanol/water partition data with the PAMPA-BBB results, no correlation could be found between the log *P* or log *D*^7.4^ values and the log *P*_e_ results (R^2^=0.37 and 0.21, respectively). On the other hand, a strong correlation could be detected between the logarithm of the capacity factor (log *k*) and log *P*_e_ values (R^2^=0.97) (Fig. S2), suggesting, that the C18 sidechains of the chromatographic column represents better the biomimetic artificial membrane of PAMPA-BBB, than octanol. Plotting the values of membrane retention similarly, no significant correlation could be found either for the partition coefficient (R^2^=0.13 for log *D*^7.4^, R^2^=0.31 for log *P*), or the membrane retention and log *k* (R^2^=0.66).
